# Technical Feasibility and Early Outcomes of Anatomical Laparoscopic Female Radical Cystectomy with Round Ligament Fixation to Prevent Vaginal Vault Prolapse

**DOI:** 10.3390/medicina62020324

**Published:** 2026-02-05

**Authors:** Christos Zabaftis, Filippos Nikitakis, Nikolaos Grivas, Athanasios Bouchalakis, Maria Chalkidou, Smaragda Tsela, Sotiria Tsogka, Markos Karavitakis

**Affiliations:** 2nd Department of Urology, Lefkos Stavros–The Athens Clinic, Sisini 1-3, 11528 Athens, Greece; zabaftisc@gmail.com (C.Z.); nikitakisf@gmail.com (F.N.); mpouxalakisth@gmail.com (A.B.); mariachalk@hotmail.com (M.C.); smatsela8@yahoo.gr (S.T.); sissytsogka@yahoo.gr (S.T.); markoskaravitakis@yahoo.gr (M.K.)

**Keywords:** radical cystectomy, vaginal prolapse, bladder cancer, laparoscopy, minimally invasive surgery

## Abstract

*Background and Objectives*: Vaginal vault prolapse is a known complication following anterior pelvic exenteration in women undergoing radical cystectomy. The aim of this study is to evaluate the feasibility, safety, and early outcomes of a novel anatomical approach for preventing vaginal vault prolapse after radical cystectomy. This study introduces a standardized laparoscopic technique that utilizes round ligament preservation and fixation to provide anatomical support to the vaginal apex. *Materials and Methods*: This study is a retrospective analysis of prospectively collected data from a single center, including thirteen female patients with uterus and adnexa in situ who underwent laparoscopic radical cystectomy with bilateral round ligament fixation to the vaginal cuff. The round ligaments were mobilized and sutured without tension. Vaginal closure was performed with barbed sutures. *Results*: No intraoperative complications occurred. At a median follow-up of 18.2 months, no cases of vaginal vault prolapse or dehiscence were observed. One patient experienced transient pelvic discomfort. *Conclusions*: This is the first report of a standardized mesh-free approach for vaginal apex support during laparoscopic anterior exenteration. The technique is feasible, safe, and may reduce postoperative prolapse risk.

## 1. Introduction

Radical cystectomy with anterior pelvic exenteration in female patients involves en bloc removal of the bladder, uterus, adnexa, and upper vagina. An essential part of the vaginal support is the suspension of the apex to the uterosacral–cardinal ligament complex [1]. While effective oncologically, radical cystectomy disrupts this native pelvic support and may lead to vaginal vault prolapse or, less commonly, vaginal evisceration [2,3].

According to the International Continence Society, vaginal vault prolapse is defined as the descent of the vaginal cuff scar below a point that is 2 cm less than the total vaginal length above the plane of the hymen [4]. This condition affects the women’s quality of life, as it can be associated with symptoms related to urinary, anorectal, and sexual function [5]. These symptoms include a sensation of vaginal bulging and heaviness, which gradually worsens throughout the day, voiding dysfunction with slow urinary stream, recurrent urinary tract infections, daytime frequency, nocturia, urgency, and stress incontinence. Coital dysfunction can present symptoms such as dyspareunia, difficulty achieving penetration, laxity of the vagina, and low self-esteem [6].

Postoperative prolapse has been reported in 6% to 23% of women, with the largest cohort showing a 10% incidence [7]. In contrast, studies in which the anterior vaginal wall, pubo-urethral ligaments, and internal genital organs are preserved, and minimal dissection is performed at the vaginal apex, report no cases of postoperative prolapse [8,9]. Vaginal evisceration, although rare, has been described in up to 7% of women after laparoscopic cystectomy, requiring urgent reoperation [10,11].

These complications are often difficult to manage, frequently requiring complex reconstructive surgery with variable outcomes [12,13]. The surgical options for pelvic organ prolapse repair after radical cystectomy include transabdominal or transvaginal suspension of the prolapsed vagina, with or without the use of mesh, or colpocleisis [1]. Despite their clinical significance, few techniques have been described for their prevention, particularly in the context of minimally invasive surgery.

In this study, we describe a stepwise laparoscopic technique for round ligament fixation to the vaginal cuff, aiming to provide tension-free, anatomical, and mesh-free support to the vaginal apex. We evaluate the feasibility, safety, and early functional outcomes of this approach as a potential preventive strategy

## 2. Materials and Methods

Since 2021, we have systematically implemented the round ligament fixation technique described herein in all female patients undergoing laparoscopic radical cystectomy with anterior pelvic exenteration at our institution. This study is a retrospective analysis of prospectively collected data from a single center and includes the first 13 consecutive women treated with this approach between January 2021 and April 2025.

### 2.1. Inclusion Criteria

Eligible patients met the following criteria:

- Presence of uterus and adnexa;
- Indication for radical cystectomy with removal of gynecologic organs (e.g., muscle-invasive bladder cancer, high-risk non-muscle-invasive disease);
- Preoperative confirmation of absence of pelvic floor disorders or preexisting vaginal prolapse.

The study was conducted with the understanding and informed consent of all patients. The consent form for participation was distributed to all participants and signed. The study was approved by the local Institutional Ethics Committee and carried out in accordance with the guidelines of the Declaration of Helsinki.

### 2.2. Surgical Technique Overview

#### 2.2.1. Step 1: Patient Positioning and Trocar Placement

The patients were placed in the low lithotomy position to allow access to the vagina, rectum, and perineum. The skin was prepared from the nipples to the mid-thigh, including a meticulous vaginal preparation. After the placement of drapes, an 18-Fr urethral catheter was inserted. A five-trocar transperitoneal approach was utilized in all cases. The camera port was placed just above the umbilicus. Following the establishment of the pneumoperitoneum, four working trocars were placed under endoscopic vision in a fan-shaped configuration. A steep Trendelenburg position was employed to facilitate the displacement of the small bowel from the pelvis.

#### 2.2.2. Step 2: En Bloc Resection

Standard laparoscopic anterior pelvic exenteration was performed, including removal of the bladder, uterus, adnexa, and upper vaginal vault. An extended pelvic lymphadenectomy was performed in all cases.

#### 2.2.3. Step 3: Round Ligament Identification and Mobilization

**Figure 1:**
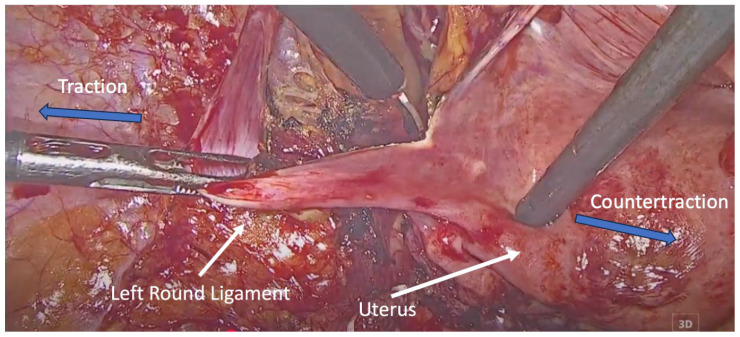
Dissection of the left round ligament. Blue arrows indicate the direction of traction and countertraction applied by the surgical instruments during dissection of the round ligament.

Following resection, the round ligaments were bilaterally identified at their entry into the deep inguinal rings. Each ligament was dissected and mobilized proximally up to the internal inguinal ring, preserving its fibromuscular integrity (Figure 1).

#### 2.2.4. Step 4: Vaginal Cuff Closure and Round Ligament Fixation

**Figure 2:**
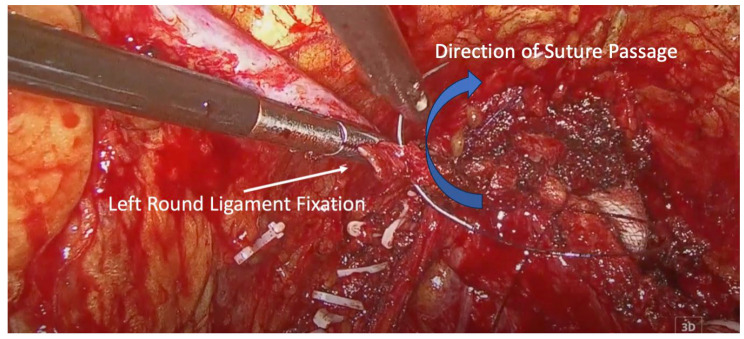
Left round ligament fixation. Blue arrow indicates the direction of suture passage through the round ligament.

**Figure 3:**
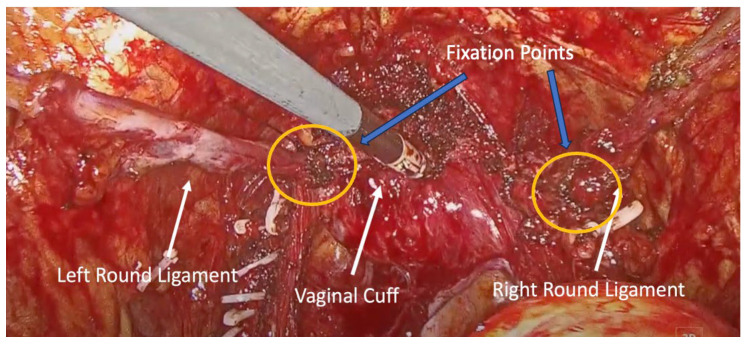
Final view of the round ligaments attached to the lateral vaginal wall. Blue arrows indicate the fixation points of the round ligaments to the vaginal cuff.

The vaginal cuff was closed using two V-Loc^TM^ (Medtronic, Minneapolis, MN, USA) barbed sutures, starting from the midline and extending laterally. At the end of closure, each suture was used to anchor the corresponding round ligament to the lateral vaginal cuff, creating a tension-free autologous sling for apical support, without mesh or foreign material (Figure 2 and Figure 3).

### 2.3. Urinary Diversion

All patients underwent intracorporeal urinary diversion, as follows:

- Three patients received an orthotopic neobladder;
- Eight patients underwent an ileal conduit (Bricker);
- Two patients had cutaneous ureterostomies.

### 2.4. Outcome Measures

- Feasibility: Identification, mobilization, and fixation of the round ligaments.
- Intraoperative metrics: Additional time required for ligament fixation.
- Complications: Intraoperative and postoperative events.
- Pelvic floor integrity: Presence of vaginal prolapse or dehiscence.
- Subjective symptoms: Patient-reported pelvic tension or pain.

### 2.5. Follow-Up

Patients were evaluated at 1, 3, 6, 12, and 18 months postoperatively with pelvic examination and standardized symptom questionnaires.

## 3. Results

A total of 13 female patients underwent laparoscopic radical cystectomy with anterior exenteration and round ligament fixation between January 2021 and April 2025. The median age was 63 years (range: 48–74). Indications included muscle-invasive bladder cancer (MIBC) in nine patients and recurrent high-risk non-muscle-invasive bladder cancer (NMIBC) in four patients. Urinary diversion was performed as an ileal conduit in eight patients, an orthotopic neobladder in three patients, and cutaneous ureterostomies were performed in two patients. Five patients (38%) had an ASA score ≥ 3 (Table 1).

Round ligament fixation was successfully completed in all 13 cases without intraoperative complications. The median additional operative time for the fixation step was 9.2 min (range: 7–12 min). There were no adverse events directly related to ligament mobilization or suturing. Overall operative time and blood loss were comparable to institutional benchmarks for female laparoscopic anterior exenteration procedures.

With a median follow-up of 18.2 months, no cases of vaginal vault prolapse or cuff dehiscence were observed. One patient reported transient pelvic discomfort during the early postoperative period, which resolved spontaneously within 6 weeks. No fixation-related complications were reported. These findings support the feasibility, safety, and anatomical integrity of the proposed technique in the short- to mid-term postoperative period.

## 4. Discussion

Anterior pelvic exenteration in women inherently disrupts the natural apical support mechanisms of the vagina. The removal of the uterus, adnexa, and upper vaginal vault eliminates key ligamentous and fascial structures that contribute to pelvic stability. Conventional reconstructive options, such as uterosacral ligament suspension or sacrocolpopexy, are often not feasible in a minimally invasive setting or require synthetic mesh and extensive retroperitoneal dissection.

Uterosacral ligament suspension and sacrospinous ligament fixation are two native-tissue suspension techniques with high reported success rates, approximately 90% for uterosacral suspension and 88–90% for sacrospinous fixation. However, both procedures are associated with recurrence rates of approximately 10% [14,15]. In addition, uterosacral ligament suspension, particularly the vaginal approach, has been associated with ureteral injury rates of up to 11%, as well as cases of de novo stress urinary incontinence, urinary retention, and neuropathic pain related to suture placement [16,17]. Sacrospinous ligament fixation is similarly associated with neuropathy due to extensive vaginal dissection, dyspareunia, and significant vaginal scarring [15]. Another limitation of sacrospinous fixation is that it may not be feasible in women with shortened vaginal length after hysterectomy [14]. Round ligament fixation, demonstrating low recurrence and minimal complication rates in this study, may offer a preferable alternative to these techniques in the context of laparoscopic anterior exenteration.

Several studies have demonstrated that the prophylactic suspension of the vaginal stump at the time of radical cystectomy can reduce the risk of pelvic organ prolapse and improve postoperative quality of life [18,19]. However, in most of those studies, a synthetic mesh was utilized, which raises concerns regarding potential complications, including mesh erosion, pain, and mesh-related infection [20]. Mesh-based approaches, such as laparoscopic sacrocolpopexy, are highly effective, with reported success rates of up to 94% and a recurrence rate of 6% [14]. Nevertheless, the effectiveness of these approaches is diminished by concerns regarding mesh-associated complications, with the Colpopexy and Urinary Reduction Efforts (CARE) trial highlighting an estimated mesh-related complication rate of 10.5% [21].

The use of autologous rectus fascia graft for the treatment of pelvic organ prolapse has been reported in the literature, suggesting that the use of native tissues may be a safer option, especially in patients with ileal neobladder formation [22]. The use of rectus fascia grafts for the treatment of pelvic organ prolapse has demonstrated success rates ranging from 87 to 100% and, as a non-mesh alternative, mitigates mesh-related complications. However, this approach has been associated with increased patient morbidity and longer operation times, both related to graft harvesting, as well as an increased risk of incisional hernias [23,24,25].

The round ligament, although traditionally considered of limited functional importance, is consistently preserved during anterior exenteration and may serve as a reliable autologous structure for apical support. Our technique incorporates round ligament fixation to the vaginal cuff using the same barbed sutures applied for vault closure, without additional dissection, foreign material, or operative burden. The added operative time was minimal (median 9.2 min), and there were no intraoperative complications or fixation failures.

Notably, none of the 13 patients in our series developed vaginal vault prolapse or cuff dehiscence during follow-up. This is of clinical relevance, considering that reported prolapse rates following radical cystectomy (RC) range from 6% to 23%, with the largest study documenting a 10% incidence [7]. In contrast, studies reporting no prolapse emphasized preservation of the anterior vaginal wall, pubo-urethral ligaments, and limited apical dissection as protective factors [8,9]. Conversely, the absence of anterior fixation of the neobladder to the pubis or Cooper’s ligament has been associated with prolapse and urinary retention [26].

In addition to prolapse, vaginal dehiscence and evisceration represent rare but serious complications. In one retrospective study of 100 women undergoing laparoscopic RC, 7% required emergency reoperation for bowel evisceration through the vaginal cuff [10]. A multifactorial etiology has been suggested for vaginal dehiscence and evisceration during minimally invasive procedures. Contributing factors include the surgical technique (extent of vaginal resection, use of energy, technical challenges in vaginal cuff suturing), patient factors (age, vaginal atrophy), and the lack of reinforcement techniques (two-layer cuff closure, securing adequate tissue edges for suturing, bidirectional barbed suture for cuff closure) [10,27].

Organ-sparing cystectomy is increasingly performed in carefully selected women to preserve reproductive and sexual function. In this approach, the supporting structures, including the round, infundibulopelvic, and broad ligaments, are preserved, along with the female reproductive organs, in order to prevent prolapse [28]. However, the potential risk of compromised oncologic control should always be taken into consideration. This technique is not always feasible, particularly in cases of bladder neck involvement or high-stage tumors [29,30]. In such patients, native tissue-based suspension, such as round ligament fixation, may offer a simple, reproducible, and mesh-free solution to reinforce pelvic support and potentially reduce postoperative morbidity.

As a novel operative technique, round ligament fixation following radical cystectomy requires a certain learning phase. Therefore, outcomes during the early learning period of less experienced laparoscopic surgeons may differ from those reported in this study. However, this technique does not require specialized equipment or advanced laparoscopic expertise, as it is based on standard laparoscopic skills such as intracorporeal suturing and dissection of anatomical structures. Consequently, surgeons with varying levels of laparoscopic experience, but with adequate competence in fundamental laparoscopic skills, would likely be capable of adopting this surgical approach.

One important parameter of this study that should be discussed is the heterogeneity of urinary diversion types. Although the consistency of surgical execution ensured comparable surgical outcomes, not all patients in the study received the same type of urinary diversion. It is well known that each urinary diversion is characterized by a specific morbidity and complication profile, with the choice of diversion depending on patient preferences, performance status, life expectancy, oncological control, and surgeon experience [31]. This heterogeneity, combined with the small sample size, may represent a potential confounder, particularly through its influence on pelvic floor dynamics and postoperative symptom perception.

Our study is limited by the small sample size and short-to-midterm follow-up, rendering the outcomes preliminary and highlighting the need for larger, multicenter studies with longer follow-up to further evaluate these findings. Additionally, the outcomes of the study may be affected by the fact that the postoperative evaluation of the patients did not include the use of a validated pelvic organ prolapse quantification system or standardized pelvic floor assessment tool, but rather relied on clinical examination and symptom questionnaires. Moreover, functional outcomes related to sexual function and quality of life are discussed but not systematically measured in this study. Future prospective comparative studies are needed to validate the role of this technique in routine anterior exenteration protocols and to systematically assess its impact on long-term outcomes, including quality of life and sexual function, using data from validated assessment tools.

## 5. Conclusions

Round ligament fixation to the vaginal cuff is a simple, anatomical, and mesh-free technique that can be safely integrated into laparoscopic anterior pelvic exenteration. In our series, the method was feasible, added minimal operative time, and was associated with excellent short- to mid-term outcomes, with no cases of prolapse, dehiscence, or fixation-related complications. Given its ease of application and native tissue approach, this technique may serve as a valuable adjunct in patients for whom genital organ preservation is not feasible. Further prospective studies are warranted to validate its long-term efficacy and potential role in standard surgical protocols.

## Figures and Tables

**Table 1:** Patient Characteristics.

Variable	Value
Median age	63 years (range: 48–74)
Indication	MIBC ^1^ (*n* = 9), recurrent NMIBC ^2^ (*n* = 4)
Urinary diversion	Ileal conduit (*n* = 8), neobladder (*n* = 3), cutaneous ureterostomies (*n* = 2)
ASA ^3^ score ≥ 3	5/13

^1^ MIBC: muscle-invasive bladder cancer; ^2^ NMIBC: non-muscle-invasive bladder cancer; ^3^ ASA: American Society of Anesthesiologists.

## Data Availability

The data that support the findings of this study are available from the corresponding author upon reasonable request.

## References

[1] Voigt M., Hemal K., Matthews C. (2019). Influence of simple and radical cystectomy on sexual function and pelvic organ prolapse in female patients: A scoping review of the literature. Sex. Med. Rev..

[2] Wenk M.J., Westhoff N., Liedl B., Michel M.S., Grüne B., Kriegmair M.C. (2023). Evaluation of sexual function and vaginal prolapse after radical cystectomy in women: A study to explore an under-evaluated problem. Int. Urogynecol. J..

[3] Lin F.C., Medendorp A., Van Kuiken M., Mills S.A., Tarnay C.M. (2019). Vaginal dehiscence and evisceration after robotic-assisted radical cystectomy: A case series and review of the literature. Urology.

[4] Abrams P., Cardozo L., Fall M., Griffiths D., Rosier P., Ulmsten U., Van Kerrebroeck P., Victor A., Wein A. (2002). The standardisation of terminology of lower urinary tract function: Report from the Standardisation Sub-committee of the International Continence Society. Neurourol. Urodyn..

[5] Uzoma A., Farag K.A. (2009). Vaginal vault prolapse. Obstet. Gynecol. Int..

[6] Robinson D., Thiagamoorthy G., Cardozo L. (2018). Post-hysterectomy vaginal vault prolapse. Maturitas.

[7] Richter L.A., Egan J., Alagha E.C., Handa V.L. (2021). Vaginal complications after radical cystectomy for bladder cancer: A systematic review. Urology.

[8] Chang S.S., Cole E., Cookson M.S., Peterson M., Smith J.A. (2002). Preservation of the anterior vaginal wall during female radical cystectomy with orthotopic urinary diversion: Technique and results. J. Urol..

[9] Roshdy S., Senbel A., Khater A., Farouk O., Fathi A., Hamed E., Denewer A. (2016). Genital sparing cystectomy for female bladder cancer and its functional outcome: A seven years’ experience with 24 cases. Indian J. Surg. Oncol..

[10] Kanno T., Ito K., Sawada A., Saito R., Kobayashi T., Yamada H., Inoue T., Ogawa O. (2019). Complications and reoperations after laparoscopic radical cystectomy in a Japanese multicenter cohort. Int. J. Urol..

[11] Schröder C., Plöger R., Knüpfer S., Padrón L.T., Ralser D.J., Otten L.A., Egger E.K., Mustea A., Könsgen D. (2024). Anterior enterocele after cystectomy: Case report and review of the literature. Arch. Gynecol. Obstet..

[12] Fort M.W., Carrubba A.R., Chen A.H., Pettit P.D. (2020). Transvaginal enterocele and evisceration repair after radical cystectomy using porcine xenograft. Female Pelvic Med. Reconstr. Surg..

[13] Stav K., Dwyer P.L., Rosamilia A., Lim Y.N., Alcalay M. (2009). Transvaginal pelvic organ prolapse repair of anterior enterocele following cystectomy in females. Int. Urogynecol. J..

[14] Vermeulen C.K.M., Schuurman B., Coolen A.L.W.M., Meijs-Hermanns P.R., van Leijsen S.A.L., Veen J., Bongers M.Y. (2023). The effectiveness and safety of laparoscopic uterosacral ligament suspension: A systematic review and meta-analysis. Int. J. Obstet. Gynaecol..

[15] Zhang W., Cheon W.C., Zhang L., Wang X., Wei Y., Lyu C. (2022). Comparison of the effectiveness of sacrospinous ligament fixation and sacrocolpopexy: A meta-analysis. Int. Urogynecol. J..

[16] Barber M.D., Visco A.G., Weidner A.C., Amundsen C.L., Bump R.C. (2000). Bilateral uterosacral ligament vaginal vault suspension with site-specific endopelvic fascia defect repair for treatment of pelvic organ prolapse. Am. J. Obstet. Gynecol..

[17] Turner L.C., Lavelle E.S., Shepherd J.P. (2016). Comparison of complications and prolapse recurrence between laparoscopic and vaginal uterosacral ligament suspension for the treatment of vaginal prolapse. Int. Urogynecol. J..

[18] Życzkowski M., Muskała B., Kaletka Z., Bryniarski P., Nowakowski K., Bogacki R., Paradysz A. (2015). Sacrocolpopexy with polypropylene tape as a valuable surgical modification during cystectomy with orthotopic ileal bladder: Functional results. Biomed. Res. Int..

[19] Törzsök P., Bauer S., Forstner R., Sievert K.-D., Janetschek G., Zimmermann R. (2016). Laparoscopic radical cystectomy and ileal neobladder for muscle-invasive bladder cancer in combination with one-stage prophylactic laparoscopic sacrospinal fixation to avoid future pelvic organ prolapse. J. Endourol. Case Rep..

[20] Littlejohn N., Cohn J.A., Kowalik C.G., Kaufman M.R., Dmochowski R.R., Reynolds W.S. (2017). Treatment of pelvic floor disorders following neobladder. Curr. Urol. Rep..

[21] Nygaard I., Brubaker L., Zyczynski H.M., Cundiff G., Richter H., Gantz M., Fine P., Menefee S., Ridgeway B., Visco A. (2013). Long-term outcomes following abdominal sacrocolpopexy for pelvic organ prolapse. JAMA.

[22] Abraham N., Quirouet A., Goldman H.B. (2016). Transabdominal sacrocolpopexy with autologous rectus fascia graft. Int. Urogynecol. J..

[23] Matak L., Baekelandt J., Šimičević M., Matak M., Mikuš M., Orešković S. (2024). Comparison between fascia lata and rectus fascia in treatment of pelvic organ prolapse: A systematic review. Arch. Gynecol. Obstet..

[24] Sharifiaghdas F. (2022). Autologous rectus fascia graft in the treatment of high-stage apical vaginal prolapse: Preliminary results of a new surgical approach with native tissue. Int. Urol. Nephrol..

[25] Wang R., Reagan K., Boyd S., Tulikangas P. (2022). Sacrocolpopexy using autologous rectus fascia: Cohort study of long-term outcomes and complications. Int. J. Obstet. Gynaecol..

[26] Badawy A.A., Abolyosr A., Mohamed E.R., Abuzeid A.M. (2011). Orthotopic diversion after cystectomy in women: A single-centre experience with a 10-year follow-up. Arab J. Urol..

[27] Cronin B., Sung V.W., Matteson K.A. (2012). Vaginal cuff dehiscence: Risk factors and management. Am. J. Obstet. Gynecol..

[28] Niver B.E., Daneshmand S., Satkunasivam R. (2015). Female reproductive organ-sparing radical cystectomy: Contemporary indications, techniques and outcomes. Curr. Opin. Urol..

[29] Ali-El-Dein B., Mosbah A., Osman Y., El-Tabey N., Abdel-Latif M., Eraky I., Shaaban A. (2013). Preservation of the internal genital organs during radical cystectomy in selected women with bladder cancer: A report on 15 cases with long-term follow-up. Eur. J. Surg. Oncol..

[30] Lavallée E., Dovey Z., Pathak P., Dey L., Koskela L.R., Hosseini A., Waingankar N., Mehrazin R., Sfakianos J., Hosseini A. (2021). Functional and oncological outcomes of female pelvic organ-preserving robot-assisted radical cystectomy. Eur. Urol. Open Sci..

[31] Barone B., Napolitano L., Reccia P., Calace F.P., De Luca L., Olivetta M., Stizzo M., Rubinacci A., Della Rosa G., Lecce A. (2024). Advances in urinary diversion: From cutaneous ureterostomy to orthotopic neobladder reconstruction—A comprehensive review. J. Pers. Med..

